# Grafted neural stem cells show lesion-specific migration in radiation-injured rat brains

**DOI:** 10.1039/c7ra10151a

**Published:** 2018-02-05

**Authors:** Shou-Min Bai, Qiong Wang, Xiao-Li Yu, Ting Chen, Jin Yang, Jun-Tian Shi, Robert Y. L. Tsai, Hai Huang

**Affiliations:** Department of Radiation Oncology, Sun Yat-Sen Memorial Hospital, Sun Yat-Sen University Guangzhou 510120 People's Republic of China smbai999@163.com yxlyxl522@163.com chent76@mail3.sysu.edu.cn yangj286@mail2.sysu.edu.cn shijuntian@126.com; Department of Urology, Sun Yat-Sen Memorial Hospital, Sun Yat-Sen University Guangzhou 510120 People's Republic of China wangqiong9088@126.com huanghai257@126.com +86-13711502580; Institute of Biosciences and Technology, Texas A&M University Health Science Center Houston Texas 77030 USA rtsai@ibt.tamhsc.edu +1-713-677-7690; Department of Molecular and Cellular Medicine, Texas A&M University Health Science Center College Station Texas 77843 USA

## Abstract

Neural stem cells (NSCs) exhibit preferential homing toward some types of brain lesion, but their migratory property during radiation brain injury (RBI) remains unexplored. Here, we use the superparamagnetic iron oxide (SPIO)-labeled magnetic resonance imaging (MRI) technology to determine the migration of transplanted NSCs in two partial RBI models in real time, created by administering 30–55 Gy of radiation to the right or posterior half of the adult rat brain. SPIO-labeled NSCs were stereotactically grafted into the uninjured side one week after RBI. The migration of SPIO-labeled NSCs in live radiation-injured brains was traced by MRI for up to 28 days after engraftment and quantified for their moving distances and speeds. A high labeling efficiency (>90%) was achieved by incubating NSCs with 100 μg ml^−1^ of SPIO for 12–24 hours. Upon stereotactic transplantation into the healthy side of the brain, SPIO-labeled NSCs were distinctively detected as hypointense signals on T2-weighted images (T2WI), showed sustained survival for up to 4 weeks, and exhibited directional migration to the radiation-injured side of the brain with a speed of 86–127 μm per day. The moving kinetics of grafted NSCs displayed no difference in brains receiving a high (55 Gy) *vs.* moderate (45 Gy) dose of radiation, but was slower in the right RBI model than in the posterior RBI model. This study shows that NSCs can be effectively labeled by SPIO and traced *in vivo* by MRI, and that grafted NSCs exhibit directional migration toward RBI sites in a route-dependent but radiation dose-independent manner.

## Abstract 2

Neural stem cells (NSCs) exhibit preferential homing toward some types of brain lesion, but their migratory property during radiation brain injury (RBI) remains unexplored.

## Graphical abstract

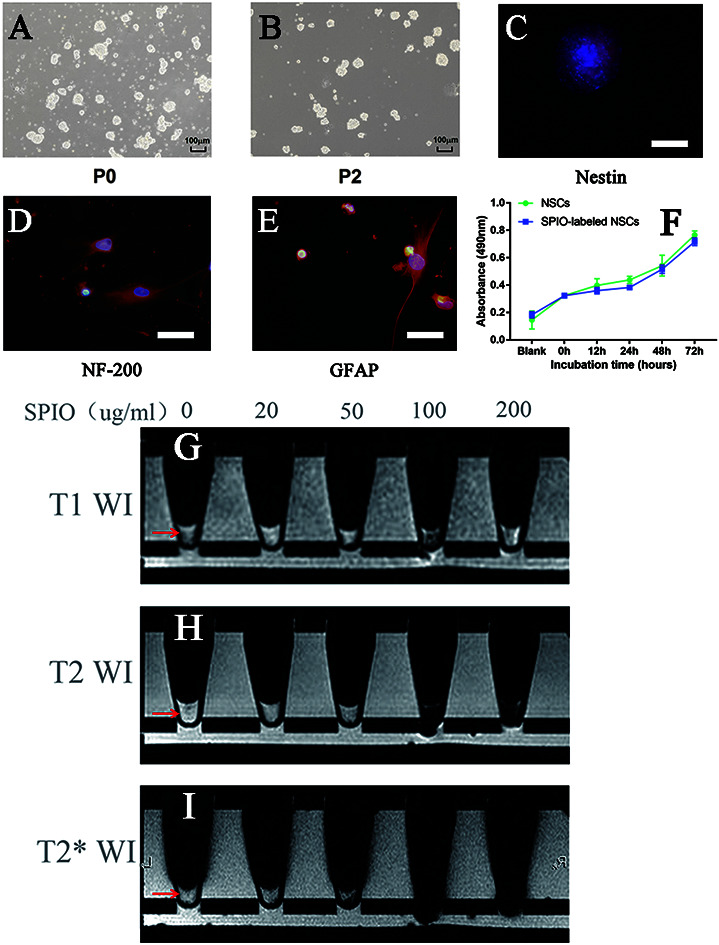

## Introduction

Radiotherapy has become the preferred method of treatment for many types of tumors in modern medicine, especially those in the head and neck region.^1,2^ It provides clear benefits of improved life expectancy and quality for cancer survivors. To date, the three-year survival rate of nasopharyngeal carcinoma after radiotherapy has reached 80–90%.^1^ However, along with the prolonged survival of radiation-treated cancer patients, the incidence of radiation-induced complications has also increased and now become a major health issue.^3,4^ Radiation brain injury (RBI) frequently occurs in patients receiving radiation treatment for head and neck tumors. It can lead to seizure, memory loss, and permanent focal neurological deficits,^5,6^ and accounts for 65% of the total death caused by post-radiation complications.^7^ To date, the therapeutic options for RBI treatment remain limited due to the lack of robust regeneration in the adult brain. As a result, there is an urgent need to explore the mechanism of radiation encephalopathy and to find new therapeutic modalities for treatment.

Recent advance in regenerative medicine has suggested that transplantation of neural stem cells (NSCs) may provide a restorative cure for brain lesions in animal models.^8–10^ NSCs are capable of self-renewal and multi-lineage differentiation^11^ and have been tested as a novel cell-based therapy in various rodent models of neurological diseases, including the spinal cord injury, Parkinson's disease, and cerebral ischemia.^12–14^ It was reported that transplanted NSCs can survive and differentiate into functional neurons, astrocytes and oligodendrocytes in the disease animal models,^11–14^ some of which even exhibit behavioral or histological improvement.^12–19^ The latter finding raises the hope that NSCs may be used to treat RBI as a neuroprotective or reparative therapy or as a drug delivery vehicle. The exact mechanism by which NSC-based therapy works is not entirely clear. It may involve the regulation of inflammatory response, neurotrophin secretion, neuronal apoptosis, focal angiogenesis, VEGF expression, and/or neurogenesis.^20,21^ One key character of NSCs in therapeutic use is their homing ability from the injection site to the lesion site. The migration of NSCs is driven by multiple factors, including chemical inducers, cell adhesion molecules, and ligand receptor interaction.^22,23^ It has been shown that transplanted NSCs are capable of migrating to acute brain lesions caused by ischemia or trauma.^24–26^ As RBI also increases inflammation and growth factor secretion,^27^ we reason that NSCs may display a preferential tropism to the radiation-damaged brain lesion as well. So far, there have been few reports on how grafted NSCs behave in radiation-injured brains.

To address this issue in live animals, we created two rodent partial RBI models and tracked the migratory behavior of transplanted NSCs *in vivo* by the SPIO-labeled MRI technology.^28–31^ Our results support a directional migration of grafted NSCs toward radiation-induced lesions and demonstrate that the RBI coupled with the SPIO-labeled MRI technology may provide a new method for studying the migratory behavior of cells in live brains.

## Experimental design

### Animal studies

All animals were housed in the Program for Animal Resources and handled in accordance with the principles of the Guide for the Care and Use of Laboratory Animals as specified by the United States Public Health Service's Policy on Humane Care and Use of Laboratory Animals. All procedures were approved by the Institutional Animal Care and Use Committee “Ethics Committee of Experimental Animal Center of Sun Yat-Sen University”.

### NSC culture and validation

The preparation of NSCs follows the same procedure described previously.^32^ In short, NSCs were isolated from the periventricular regions of 1 day-old Sprague Dawley rats, maintained in suspension culture as neurospheres in DMEM/F12 medium supplemented with B27 (2%), FGF2 (20 ng ml^−1^), EGF (20 ng ml^−1^), and heparin (5 ng ml^−1^), and passaged at a 1 : 2 or 1 : 3 ratio. Primary NSCs were stained with anti-nestin and anti-Tuj1 antibodies following standard immunostaining procedures.^32^ To demonstrate their multi-lineage potential, NSCs were induced to differentiate into neurons or astrocytes by the differentiation conditions as described in the previously published methods,^33^ and stained with anti-NF-200 antibody for neurons or anti-GFAP antibody for astrocytes.

### SPIO labeling and MRI scanning

To determine the SPIO-labeling efficiency, NSCs (1 × 10^5^) were incubated with different concentrations of SPIO (20 μg ml^−1^, 50 μg ml^−1^, 100 μg ml^−1^, or 200 μg ml^−1^), protamine sulfate (4 μg ml^−1^), and serum-free medium (0.5 ml) in a final volume of 2 ml. Labeled NSCs were then fixed in 4% paraformaldehyde, stained in Prussian blue for 30 minutes, and counter-stained in 0.5% neutral red for 1 minute. The SPIO-labeling rate was calculated by counting the percentage of labeled cells in 30 high-power fields. To determine the MR intensity, NSCs were incubated with SPIO for 12 hours, dissociated into single cell suspension, frozen in ethylene propylene tubes coated with 50 μl of 4% gelatin, and subjected to MR imaging.

### Cell viability and proliferation assay

To determine the viability of SPIO-labeled NSCs, cells were dissociated, stained with 0.4% trypan blue (TB), and quantified for the non-TB-labeled cell percentage. To determine cell proliferation, NSCs were plated at the 1 × 10^4^ per well density in 96-well plates, mixed with PRO and SPIO, cultured for 0, 12, 24, 48 or 72 hours in duplicate samples, and measured for the amounts of viable cells at the end of each time point by the 3-(4,5-dimethylthiazol-2-yl)-2,5-diphenyl tetrazolium bromide (MTT) assay (Sigma, USA). In control groups, NSCs were incubated with PRO without SPIO. For MTT assay, cells were incubated with 200 μl MTT (5 mg ml^−1^) at 37 °C for 4 hours and lysed in 150 μl DMSO for 5 min. Optical densities were measured by using the Versamax microplate reader (Molecular Devices, Sunnyvale, CA) at 490 nm.

### RBI model

The top and lateral X-ray images were taken by a simulation positioning machine (Toshiba LX-40A, Japan). Shields for the posterior and right RBI models were made. Radiation was administered by using the Siemens Primus Linear Accelerator (Siemens Healthcare, USA). The 6 MV photon was used to laterally irradiate the posterior half of the brain with a Source Skin Distance (SSD) of 60 cm, dose rate of 200 cGy min^−1^, depth of 2.5 cm, and bolus of 1 cm. The 6 MeV electron beam was used to vertically irradiate the right half of the brain with a SSD of 100 cm, dose rate of 300 cGy min^−1^, and depth of 1.5 cm. The right RBI group consisted of 6 rats receiving 30 Gy of radiation in a single dose. The posterior RBI group included 6 rats receiving 45 Gy of radiation in a single dose and 6 rats receiving 55 Gy of radiation in a single dose.

### Stereotactic transplantation and MRI scanning

NSC transplantations were performed 7 days after the radiation treatment. PRO (4 μg ml^−1^) and SPIO (100 μg ml^−1^) were premixed for 30 minutes. NSCs in 24-well plates were incubated with the PRO–SPIO mixture for 12 hours and dissociated into single cell suspension at 5 × 10^3^ cells per μl. Dissociated NSCs (5 × 10^4^ cells in 10 μl) were stereotactically transplanted into the non-irradiated side of the brain within a 10 minute injection time window using the standard stereotactic instrument (model 68001, RWD Life Science, USA). The mid-point of two ears is set as point zero. The posterior RBI group received NSC transplantation at a site 9 mm anterior, 3 mm left, and 5 mm down to point zero. The right RBI group received NSC transplantation at a site 8.5 mm anterior, 3 cm left, and 5 mm down to point zero. Control rats were transplanted with non-SPIO-labeled NSCs or the SPIO dye alone. MRI scanning was conducted at the 0, 7, 14, and 28 day time points after transplantation using a 1.5 Tesla Superconducting MR Scanner and rat-specific solenoid. MRI scanning was performed in the order of SE (spin echo) T1WI (coronal section) and TSE (turbo spin echo) T2WI (coronal and sagittal sections). The TR (repetition time), TE (echo time), NSA (number of signal accept), thickness, and voxel for T1WI are 300 ms, 15 ms, 1 time, 2 mm, and 0.3 × 0.5, respectively. The TR, TE, NSA, thickness, and voxel for T2WI are 1600 ms, 50 ms, 1 time, 2 mm, and 0.4 × 0.4, respectively. Matrix size equals 512 × 512.

### Migration analysis

The distance of directional migration was defined as the distance between the tip of the injection needle and the leading end of the low T2-signaled protrusion from the injected NSCs. The distance of NSC migration toward the lesion-opposite direction (non-directional) was measured along the same axis as but 180° to that of directional migration. Each distance was measured twice independently using the Osirix 6.4 program and averaged.

### Statistical analysis

Data were analyzed by using Statistic Package for Social Science version 16.0 and represented as mean (±sd). OD_570_ readings were compared by *t*-test. In Table 1, the intensities of SPIO signals were compared by one-way analysis of variance and q-test. Differences were considered significant if two-tailed *p*-values are less than 0.05.

**Table 1:** The MR intensities of NSCs labeled with different concentrations of SPIO^a^

Concentration	T1WI	T2WI	T2WI^b^
0 (control)	254.2 ± 28.7	364.2 ± 15.2	422.5 ± 5705
20 (μg ml^−1^)	291.8 ± 32.9	322.9 ± 22.0	438.8 ± 36.6
50 (μg ml^−1^)	273.7 ± 21.7	295.9 ± 24.0^b^	410.8 ± 53.8
100 (μg ml^−1^)	143.1 ± 36.4^c^	20.1 ± 12.0^b^	36.8 ± 22.7^b^
200 (μg ml^−1^)	275.4 ± 28.1	90.5 ± 34.1^b^	197.0 ± 75.4^b^

a Data represent mean ± sd.

b *p* < 0.01.

c *p* < 0.05.

## Results and discussion

### SPIO labeling of NSCs *in vitro* and its effect on cell survival and proliferation

**Fig. 1:**
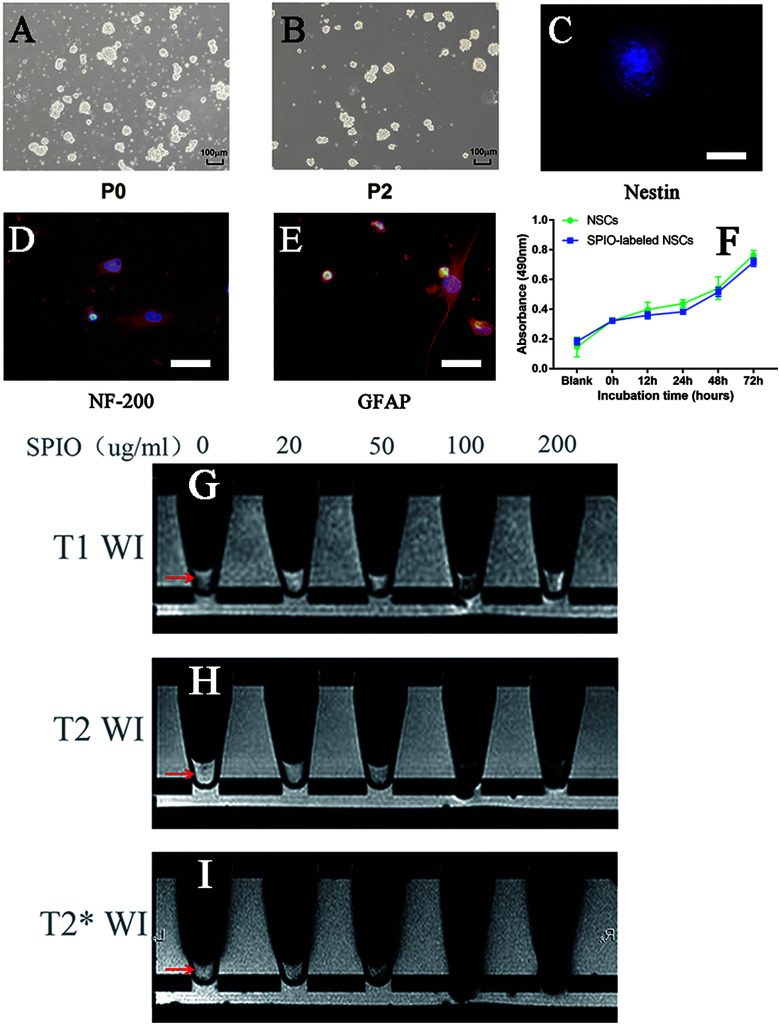
Morphology, identification and SPIO-labeling of the NSCs. (A, B) primary (P0) and passaged (P2) NSCs under 100× magnification. Scale bars show 100 μm. (C) Photomicrographs of neurospheres positive marker nestin under 200× magnification. Scale bars show 50 μm. (D) NF-200 staining of neuron in the NSCs after neuron-induction under 400× magnification. Scale bars show 25 μm. (E) GFAP staining of astrocyte in the NSCs after astrocyte-induction under 400× magnification. Scale bars show 25 μm. (F) Measurement of 100 μg ml^−1^ SPIO-labeled NSCs growth viability by MTT assay in different time points of two groups. (G–I) NSCs were labeled with 0, 20, 50, 100, or 200 μg ml^−1^ of SPIO and MR imaged in the T1-weighted (G), T2-weighted (H) or T2* sequence (I).

Primary neural cells grew mostly as colonies in amidst of single cells and flocculent tissue (Fig. 1A). After two rounds of passage selection, the suspension culture contained primarily neurospheres with relative morphological homogeneity (Fig. 1B). Immunofluorescent studies demonstrated that these neurospheres were positive for nestin (Fig. 1C) and negative for Tuj1 (data not shown), indicating that cells in the neurosphere culture were undifferentiated neural stem/progenitor cells. Under the neuronal or astrocytic differentiation condition, these undifferentiated progenitors could differentiate into NF-200-positive neurons (Fig. 1D) or GFAP-positive astrocytes (Fig. 1E), respectively, indicating that the cells in our culture could be operationally defined as NSCs based on their marker expression, multi-passage growth (self-renewal), and multi-differentiation potential.

NSCs incubated with increasing concentrations of SPIO at 20 μg ml^−1^, 50 μg ml^−1^, 100 μg ml^−1^, and 200 μg ml^−1^ for 12 hours showed respective labeling efficiencies of 91%, 93%, 97%, and 99%. The signal intensities of SPIO-labeled NSCs *in vitro* were imaged by MRI (Fig. 1G and H) and quantified to determine the best labeling condition (Table 1). Of all the concentrations tested, only the 100 μg ml^−1^ group showed a lower T1-weighted signal compared to the non-labeled control (*p* < 0.05) (Fig. 1G). By comparison, the T2-weighted (Fig. 1H) and T2* sequences (Fig. 1I) detected NSCs labeled with a wider range of SPIO concentrations, showing low T2 signals in the 50 μg ml^−1^, 100 μg ml^−1^, and 200 μg ml^−1^ groups (*p* < 0.01) and low T2* signals in the 100 μg ml^−1^ and 200 μg ml^−1^ groups (*p* < 0.01). The proliferative activities of NSCs labeled with SPIO at the 20 μg ml^−1^, 50 μg ml^−1^, 100 μg ml^−1^, or 200 μg ml^−1^ for 12 hours concentration are 98.9%, 97.9%, 97.9%, and 95.8% of that of non-labeled NSCs, respectively. The relative survival rates of SPIO-labeled NSCs are 98.8% (20 μg ml^−1^), 98.8% (50 μg ml^−1^), 95.2% (100 μg ml^−1^), and 94.0% (200 μg ml^−1^) compared to non-labeled NSCs (0 μg ml^−1^). These results showed that, of all the tested concentrations, 100 μg ml^−1^ of SPIO yields the most distinct MRI signal change in the T1-weighted, T2-weighted, and T2* sequences *in vitro*, which is in congruence with a previous report that a higher concentration of SPIO (≥100 μg ml^−1^) is necessary for *in vivo* detection by MRI.^34^ More importantly, there is no significant difference in the survival or proliferation between the control and 100 μg ml^−1^ SPIO-labeled NSCs from 0 hour up to 72 hours (Fig. 1F, *P* > 0.05), which is also consistent with the result shown in a previous study.^8^

### Creation of the posterior and right RBI models

**Fig. 2:**
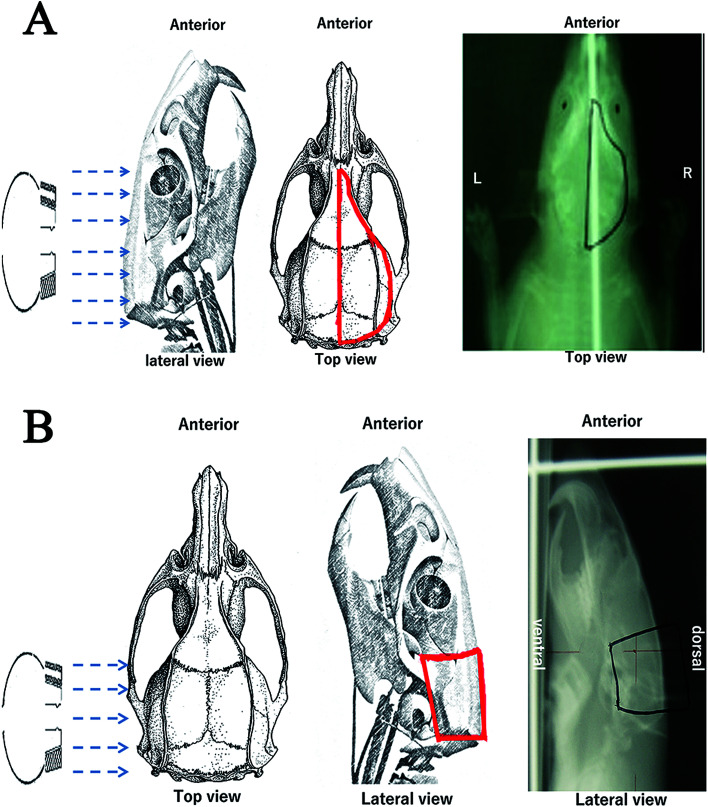
Rat models with radiation brain injury (RBI). Schematic diagrams and X-ray films of the right (A) and posterior (B) RBI models. The electron beam and irradiated regions are indicated by the blue arrows and red lines, respectively. Abbreviations: A, anterior; P, posterior; R, right; L, left; D, dorsal; V, ventral.

Due to the high radiation tolerance of rat brains, there is no standard method for preparing RBI models in rats to date. To determine the migratory properties of grafted NSCs, we created two new RBI models in rats, where 45–55 Gy and 30 Gy of radiation were administered to the posterior and right half of the brain, respectively (Fig. 2). The dose of radiation was decided based on our pilot studies that determine the maximal radiation dose without major side effects. Compared to the posterior RBI model, the right RBI model was irradiated over a larger area of the head. As a result, 45 Gy of radiation in the right RBI model created severe oral ulcer and mucositis that often led to feeding difficulty and death. We therefore chose 30 Gy instead for the right RBI model. Under these dosages, all rats survived the radiation treatment throughout the 35 day period of the study. After RBI, rats were maintained for 7 days before receiving intracranial transplantation of NSCs. The 7 day waiting period has been shown to be therapeutically favorable by a previous study.^35^ For transplantation, NSCs were injected into the healthy side of the brain using the stereotactic technique as described in the Experimental design section. About 7% of the rats receiving intracranial injection of NSCs or SPIO dye alone died within one day, which may be caused by the intracranial infection. While we might further reduce the mortality rate by injecting antibiotics post-operatively to all animals, the remaining 93% of rats had already provided enough samples for the following studies.

### Migration of SPIO-labeled NSCs in the posterior RBI model

**Fig. 3:**
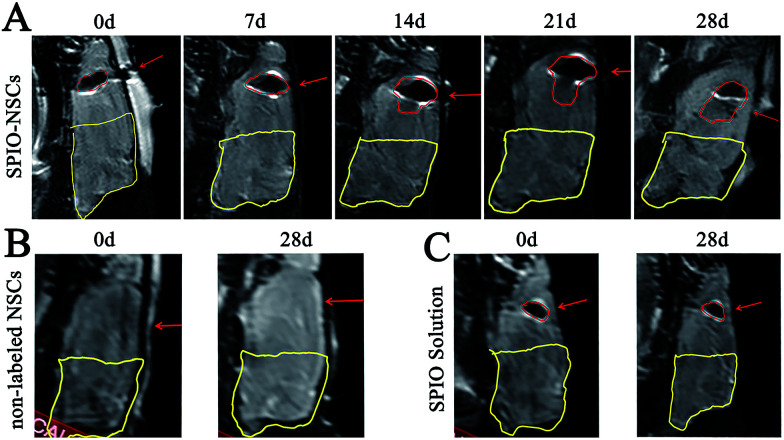
MR images of the 45 Gy posterior RBI model. (A) T2-weighted images (T2WI) of rat brains that received 45 Gy of radiation on the posterior brain and SPIO-labeled NSC transplantation. (B) T2WI of rat brains that received 45 Gy of radiation on the posterior brain and non-labeled NSC transplantation. (C) T2WI of rat brains that received 45 Gy of radiation on the posterior brain and SPIO solution injection. The injected site and hypointense signals were indicated by red arrows and red lines, respectively. The RBI lesion was circled by yellow lines. The orientation of the films follows that of Fig. 2B.

**Fig. 4:**
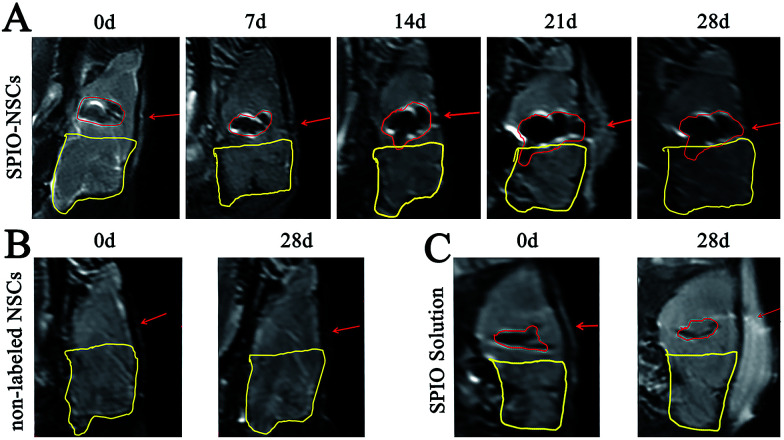
MR images of the 55 Gy posterior RBI model. (A) T2WI of rat brains that received 55 Gy of radiation on the posterior brain and SPIO-labeled NSC transplantation. (B) T2WI of rat brains that received 55 Gy of radiation on the posterior brain and non-labeled NSC transplantation. (C) T2WI of rat brains that received 55 Gy of radiation on the posterior brain and SPIO solution injection. The injected site and hypointense signals were indicated by red arrows and red lines, respectively. The RBI lesion was circled by yellow lines. The orientation of the films follows that of Fig. 2B.

**Fig. 5:**
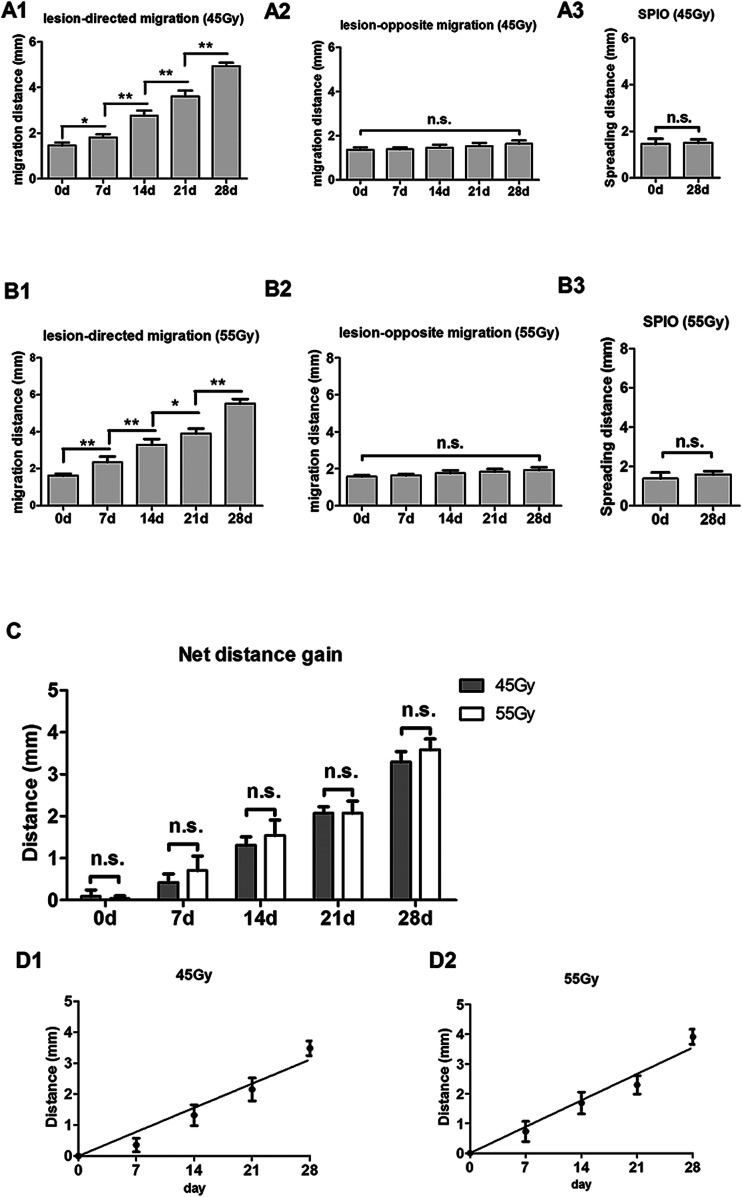
Quantification of NSC migration in the 45 Gy and 55 Gy posterior RBI models. (A) Distance of lesion-directed (A1) and lesion-opposite (A2) migration from 0 to 28 days after NSC transplantation in the 45 Gy posterior RBI model. (A3) Spreading distance of injected SPIO solution on the 0 and 28th day. (B) Distance of lesion-directed (B1) and lesion-opposite (B2) migration from 0 to 28 days after NSC transplantation in the 55 Gy posterior RBI model. (B3) Spreading distance of injected SPIO solution on the 0 and 28th day. (C) The net gain of distance between lesion-directed *vs.* lesion-opposite migration from 0 to 28 days in the 45 Gy (black bars) and 55 Gy (grey bars) models. (D) Calculation of the average speeds of migration by the slopes of the best-fit lines in the 45 Gy (D1) and 55 Gy (D2) models. Bars show mean (±sd); * and ** indicate *p* values < 0.01 and 0.001; n.s., not significant.

To track SPIO-labeled NSCs *in vivo*, we chose early-passage NSCs (P2 or P3) and the SPIO labeling time of 12 hours. Early-passage NSCs were chosen over late-passage NSCs because they were exposed to the *in vitro* stress condition for a shorter period of time and hence might better represent cells *in vivo*.^36,37^ A 12 hour labeling time was also preferred to a 48 or 72 hour labeling time for the same reason, especially if it showed the same labeling efficiency as did the 48 or 72 hour labeling. The choice of a 12 hour labeling time is also in line with what most studies have used previously.^8,38–40^ After stereotactic transplantation, the distributions of SPIO-labeled and non-labeled NSCs were imaged with 1.5 T MR every 7 days for 28 days. In the 45 Gy and 55 Gy posterior RBI models, SPIO-labeled NSCs appeared as hypointense signals on T2WI immediately after the transplantation (Fig. 3A and 4A, marked by red lines). In the early stage, grafted SPIO-labeled NSCs showed well-defined margins surrounded by edematous rims of high T2-weighted signals. The spreading of SPIO-labeled NSCs from the injection site toward the RBI site began within 7 days and continued to increase over the next 3 weeks. In the control groups, non-labeled NSCs showed no detectable signal on T2WI (Fig. 3B and 4B), and injected SPIO dye showed no discernible spreading (Fig. 3C and 4C). The distances of lesion-directed (directional) *vs.* lesion-opposite (non-directional) migration were quantitatively measured based on the criteria described in the Experimental design. Our analyses showed that the distance of RBI-directed migration of NSCs increased significantly (Fig. 5A1 and 5B1). In contrast, the distance of non-directional migration of NSCs (Fig. 5A2 and 5B2) or the migration distance of SPIO dye (Fig. 5A3 and 5B3) remained unchanged in both the 45 Gy and 55 Gy posterior RBI models. Notably, there was no statistical difference in the migrating distances or the average speed of migration for 45 Gy and for 55 Gy between the 45 Gy RBI model (111 μm per day) and the 55 Gy RBI model (127 μm per day) (Fig. 5C). These results show that grafted NSCs exhibit lesion-directed migration in the posterior RBI model, and that the movement kinetics of grafted NSCs is not affected by the dose of radiation (45 Gy *vs.* 55 Gy).

### Migration of SPIO-labeled NSCs in the right RBI model

**Fig. 6:**
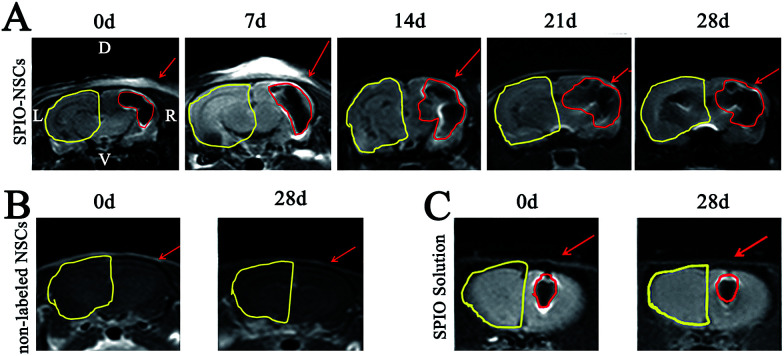
MR images of the 30 Gy right RBI model. (A) T2WI of rat brains that received 30 Gy of radiation on the right brain and SPIO-labeled NSC transplantation. (B) T2WI of rat brains that received 30 Gy of radiation on the right brain and non-labeled NSC transplantation. (C) T2WI of rat brains that received 30 Gy of radiation on the right brain and SPIO solution injection. The injected site and hypointense signals were indicated by red arrows and red lines, respectively. The RBI lesion was circled by yellow lines. Abbreviations: R, right; L, left; D, dorsal; V, ventral.

**Fig. 7:**
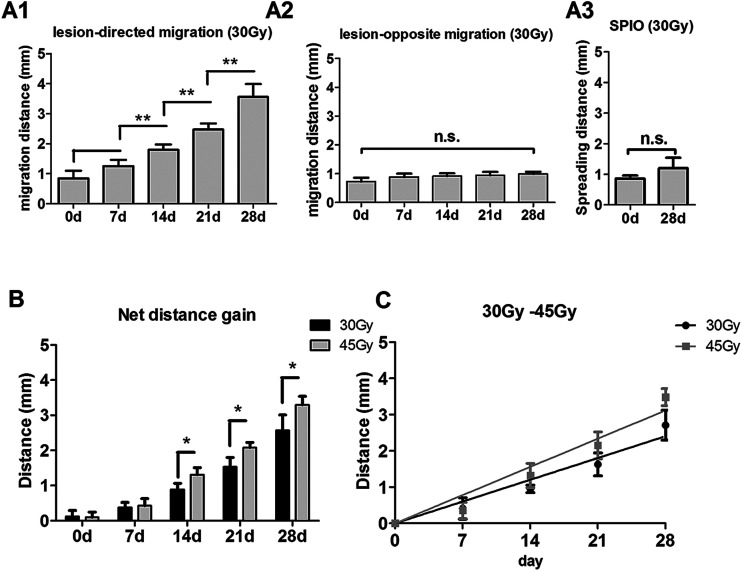
Quantification of NSC migration in the 30 Gy right RBI models. (A) Distance of lesion-directed (A1) and lesion-opposite (A2) migration from 0 to 28 days after NSC transplantation in the 30 Gy right RBI model. (A3) Spreading distance of injected SPIO solution on the 0 and 28th day. (B) The gain of distance between lesion-directed *vs.* lesion-opposite migration from 0 to 28 days in the 30 Gy right RBI (black bars) and 45 Gy posterior RBI (grey bars) models. (C) Calculation of the average speed of NSC migration in the right RBI model by the slope of the best-fit line.

In the right RBI model, SPIO-labeled NSCs were readily detected as T2 hypointense signals after the transplantation (Fig. 6A). The spreading of hypointense signals toward the lesion (left) side were also detected within 7 days and increased throughout the next 3 weeks (Fig. 6A). Similar to the posterior RBI models, non-labeled NSCs showed no detectable on T2WI (Fig. 6B), and injected SPIO dye exhibited no discernible spreading (Fig. 6C). Quantitative analyses confirmed that the distance of directional migration of NSCs (Fig. 7A1) was significantly increased, whereas the distance of non-directional migration of NSCs (Fig. 7A2) or the migration distance of SPIO dye (Fig. 7A3) remained unchanged over time. Compared to those in the 45 Gy posterior RBI model, grafted NSCs in the right RBI model began to show a slower migration on the 14th day and later, with an average speed of 86 μm per day (Fig. 7B). These results show that grafted NSCs exhibit lesion-directed migration in the right RBI model, and that the movement kinetics of grafted NSCs is significantly slower in the right RBI model compared to the posterior RBI model.

### *In vivo* tracing of grafted NSCs by SPIO labeling and MRI

Here, we show that incubation with 100 μg ml^−1^ of SPIO for 12 hours allows effective labeling of NSCs *in vitro* and accurate imaging of grafted NSCs *in vivo* by MRI for up to a month. At this concentration, SPIO labeling does not affect the survival or proliferation of NSCs to any significant extent. One thing of note is that the MRI signal of grafted NSCs shows a slight decrease over time, which is consistent with the findings reported previously.^12,41^ The decrease of SPIO signal may be caused by biological rather than technical reasons, such as cell division,^42^ cell migration,^43^ iron metabolism,^17^ and/or macrophage phagocytosis.^39^ One concern of using SPIO to track the migration of NSCs is that it may perturb the motility of NSCs. In our study, the migration of grafted NSCs toward the RBI site is clearly detected by MR imaging. Because labeled NSCs do not spread in the lesion-opposite direction and they do not migrate in healthy rat brains^25^ unless injured,^24–26^ we conclude that the direction of NSC migration is specifically pointed at the RBI site and that this movement can be readily detected by using the SPIO-labeled MRI approach. Notably, one limitation of this SPIO-MRI-based cell-tracking system is that it cannot definitively determine the absolute number of migratory cells. Further improvement on the detection sensitivity and linearity of this system is needed to resolve this issue.

### Factors affecting NSC migration in radiation-injured brains

Our RBI models also allow us to determine whether the location or severity of radiation damage may influence the migratory behavior of NSCs. We found that the hypointense signal spreads from the uninjured site to the damage site in both the right and posterior RBI models. The directional spreading of hypointense signals was detected within 1 week after the transplantation in all RBI models. In the same posterior RBI model, the migration speed of NSCs does not differ statistically in brains receiving different doses of radiation, showing average speeds of 111 μm per day and 127 μm per day in the 45 Gy and 55 Gy posterior RBI models, respectively. In contrast, the migration distance of NSCs is shorter in the right RBI model (30 Gy) than in the posterior RBI model (45 Gy). The difference in migration speed may reflect the different radiation dosages. However, since the migration speeds of NSCs in the 45 Gy *vs.* 55 Gy posterior RBI model show no difference, we reason that other factors may be in play. As different regions of the brain are connected by long projection fibers in the white mater, known as the commissure, we speculate that the difference in migration distance between NSCs grafted in the right *vs.* posterior RBI models may reflect the difference in the anatomical structures of these white mater tracts that constitute their migratory paths. In addition to the grafted location, other factors, such as the injury types and the properties of grafted NSCs and recipient brains, may also influence the migration speed of NSCs,^44^ which may explain the different migration speed of NSCs seen in this study *vs.* a previous one.^39^ The establishment of this NSC-tracking-in-RBI system allows one to further dissect these issues at the molecular level in the future.

### NSC-based therapies for RBI treatment

In this study, we choose NSCs as our research object for the following reasons. First of all, NSCs offer a broad spectrum of differentiation potential and hence may be therapeutically applicable in a wide variety of neurological diseases. In addition, the methods for NSC isolation, enrichment, and expansion have been extensively documented in the literature, including some of our previous studies.^45–47^ Furthermore, it has been shown that transplanted NSCs can survive and differentiate into functional neurons, astrocytes and oligodendrocytes in diseased animal models.^11–14^ Finally, compared to pluripotent ES or iPS cells, NSCs are partially restricted in their developmental potential along the neural lineage and therefore do not give rise to teratoma after transplantation. However, clinical application of NSC-based therapies is not without concerns. For example, allogeneic NSCs may be rejected by the host's immune system. Autologous NSCs may be immunologically compatible with the host tissue, but are difficult to obtain in enough quantify due to some practical as well as ethical issues. Furthermore, the multi-lineage potential of NSCs is a double-edged sword. On the one hand, it increases their therapeutic versatility. On the other hand, it also increases the risk of generating unwanted cell types, such as glial cells or different types of neurons.

Because every cell type (*e.g.* ES, iPS cells, tissue-resident stem cells, or partially differentiated progenitor cells) has its advantages and disadvantages, it remains to be determined which one of them will become the gold standard for cell-based therapies in the future. In addition, adipose-derived stem cells, which can differentiate into smooth muscle cells or vascular endothelial cells when transplanted into defective bladders,^37,48^ may be *trans*-differentiated into functional neurons, astrocytes and oligodendrocytes under certain conditions as well and thus provide another easily accessible source for therapy. With regard to the *in vivo* migratory behavior of these many different cell sources, since they all have their unique biological characteristics, one cannot simply transpose the findings observed in NSCs to others without careful experiment testing. It should not come as a surprise if the migratory behaviors of ES cells NSCs, and neuronal progenitors are different in the end.

## Conclusion

RBI is a major side effect of radiation therapy that still lacks standard treatment to date. The goal of this study is to create a rodent model for studying the migration of NSCs in live radiation-injured brains as a prelude to address their therapeutic value in RBI. Our model is based on a half-brain RBI design and the SPIO-labeled MRI technology. We show that NSCs can be best labeled with 100 μg ml^−1^ of SPIO for *in vivo* MR imaging. SPIO-labeled NSCs survive for up to one month after engraftment and migrate toward the lesion site in radiation-injured rat brains. The rate of NSC migration is influenced by the path of migration but not by the severity of RBI. These findings provide an initial cell biological framework for designing NSC-based therapies for RBI treatment.

## Authors' contributions

SMB conceived and designed the original study and helped drafting the manuscript. QW and TC performed NSC culture and transplantation. YLM and TC created the RBI models. SKW and JTS conducted MRI scanning and migration tests. HH and RT analyzed and interpreted the data, revised the study design, designed the figures, and wrote the manuscript. All authors have read and approved the final manuscript.

## Conflicts of interest

The authors declare no competing interest.

## Abbreviations

NSCs: Neural stem cells
SPIO: Superparamagnetic iron oxide
MRI: Magnetic resonance imaging
RBI: Radiation brain injury
TB: Trypan blue
SSD: Source skin distance
ES: Embryonic stem
iPS: Induced pluripotent stem

## Supplementary Material

## References

[cit1] Chua M. L. K., Wee J. T. S., Hui E. P., Chan A. T. C. (2016). Lancet.

[cit2] Schwartz D. L., Garden A. S. (2006). Hematol. Oncol. Clin. N. Am..

[cit3] Ding Z., Zhang H., Lv X. F., Xie F., Liu L., Qiu S., Li L., Shen D. (2018). Hum. Brain Mapp..

[cit4] Makale M. T., McDonald C. R., Hattangadi-Gluth J. A., Kesari S. (2017). Nat. Rev. Neurol..

[cit5] Mao Y. P., Zhou G. Q., Liu L. Z., Guo R., Sun Y., Li L., Lin A. H., Zeng M. S., Kang T. B., Jia W. H., Shao J. Y., Mai H. Q., Ma J. (2014). Br. J. Cancer.

[cit6] Chen J., Dassarath M., Yin Z., Liu H., Yang K., Wu G. (2011). Radiat. Oncol..

[cit7] Lee A. W., Law S. C., Ng S. H., Chan D. K., Poon Y. F., Foo W., Tung S. Y., Cheung F. K., Ho J. H. (1992). Br. J. Radiol..

[cit8] Lu L., Wang Y., Cao M., Chen M., Lin B., Duan X., Zhang F., Mao J., Shuai X., Shen J. (2017). RSC Adv..

[cit9] Hao L., Zou Z., Tian H., Zhang Y., Zhou H., Liu L. (2014). BioMed Res. Int..

[cit10] Tsai R. Y. (2018). J. Clin. Invest..

[cit11] Daadi M. M., Steinberg G. K. (2009). Regener. Med..

[cit12] Daadi M. M., Hu S., Klausner J., Li Z., Sofilos M., Sun G., Wu J. C., Steinberg G. K. (2013). Cell Transplant..

[cit13] Nudi E. T., Jacqmain J., Dubbs K., Geeck K., Salois G., Searles M. A., Smith J. S. (2015). J. Neurotrauma.

[cit14] Shi H., Song J., Yang X. (2014). Neural Regener. Res..

[cit15] Roberts T. J., Price J., Williams S. C., Modo M. (2006). Neuroscience.

[cit16] Sykova E., Jendelova P. (2007). Cell Death Differ..

[cit17] Ben-Hur T., van Heeswijk R. B., Einstein O., Aharonowiz M., Xue R., Frost E. E., Mori S., Reubinoff B. E., Bulte J. W. (2007). Magn. Reson. Med..

[cit18] Park K. I., Hack M. A., Ourednik J., Yandava B., Flax J. D., Stieg P. E., Gullans S., Jensen F. E., Sidman R. L., Ourednik V., Snyder E. Y. (2006). Exp. Neurol..

[cit19] Bulte J. W., Duncan I. D., Frank J. A. (2002). J. Cereb. Blood Flow Metab..

[cit20] Tang Y., Wang J., Lin X., Wang L., Shao B., Jin K., Wang Y., Yang G. Y. (2014). J. Cereb. Blood Flow Metab..

[cit21] Weick J. P., Liu Y., Zhang S. C. (2011). Proc. Natl. Acad. Sci. U. S. A..

[cit22] Saha B., Jaber M., Gaillard A. (2012). Front. Cell. Neurosci..

[cit23] Widera D., Kaus A., Kaltschmidt C., Kaltschmidt B. (2008). J. Cell. Mol. Med..

[cit24] Hoehn M., Kustermann E., Blunk J., Wiedermann D., Trapp T., Wecker S., Focking M., Arnold H., Hescheler J., Fleischmann B. K., Schwindt W., Buhrle C. (2002). Proc. Natl. Acad. Sci. U. S. A..

[cit25] Kim D. E., Schellingerhout D., Ishii K., Shah K., Weissleder R. (2004). Stroke.

[cit26] Tang H., Sha H., Sun H., Wu X., Xie L., Wang P., Xu C., Larsen C., Zhang H. L., Gong Y., Mao Y., Chen X., Zhou L., Feng X., Zhu J. (2013). Cell. Reprogram..

[cit27] Joo K. M., Jin J., Kang B. G., Lee S. J., Kim K. H., Yang H., Lee Y. A., Cho Y. J., Im Y. S., Lee D. S., Lim D. H., Kim D. H., Um H. D., Lee S. H., Lee J. I., Nam D. H. (2012). PLoS One.

[cit28] Bulte J. W. (2009). AJR, Am. J. Roentgenol..

[cit29] Modo M., Roberts T. J., Sandhu J. K., Williams S. C. (2004). Expert Opin. Biol. Ther..

[cit30] Lepore A. C., Walczak P., Rao M. S., Fischer I., Bulte J. W. (2006). Exp. Neurol..

[cit31] Guzman R., Uchida N., Bliss T. M., He D., Christopherson K. K., Stellwagen D., Capela A., Greve J., Malenka R. C., Moseley M. E., Palmer T. D., Steinberg G. K. (2007). Proc. Natl. Acad. Sci. U. S. A..

[cit32] Tsai R. Y., McKay R. D. (2000). J. Neurosci..

[cit33] Rietze R. L., Reynolds B. A. (2006). Methods Enzymol..

[cit34] Magnitsky S., Walton R. M., Wolfe J. H., Poptani H. (2008). Acad. Radiol..

[cit35] Shetty A. K., Turner D. A. (1996). Prog. Neurobiol..

[cit36] Xiao D., Wang Q., Yan H., Lv X., Zhao Y., Zhou Z., Zhang M., Sun Q., Sun K., Li W., Lu M. (2017). Oncotarget.

[cit37] Wang Q., Xiao D. D., Yan H., Zhao Y., Fu S., Zhou J., Wang Z., Zhou Z., Zhang M., Lu M. J. (2017). Stem Cell Res. Ther..

[cit38] Ramaswamy S., Schornack P. A., Smelko A. G., Boronyak S. M., Ivanova J., Mayer Jr J. E., Sacks M. S. (2012). NMR Biomed..

[cit39] Cromer Berman S. M., Kshitiz, Wang C. J., Orukari I., Levchenko A., Bulte J. W., Walczak P. (2013). Magn. Reson. Med..

[cit40] Chen Y. C., Wen S., Shang S. A., Cui Y., Luo B., Teng G. J. (2014). Cytotherapy.

[cit41] Karki K., Knight R. A., Shen L. H., Kapke A., Lu M., Li Y., Chopp M. (2010). Brain Res..

[cit42] Sun R., Dittrich J., Le-Huu M., Mueller M. M., Bedke J., Kartenbeck J., Lehmann W. D., Krueger R., Bock M., Huss R., Seliger C., Grone H. J., Misselwitz B., Semmler W., Kiessling F. (2005). Invest. Radiol..

[cit43] Zhang R. L., Zhang L., Zhang Z. G., Morris D., Jiang Q., Wang L., Zhang L. J., Chopp M. (2003). Neuroscience.

[cit44] Naegele J. R., Maisano X., Yang J., Royston S., Ribeiro E. (2010). Neuropharmacology.

[cit45] Meng L., Lin T., Peng G., Hsu J. K., Lee S., Lin S. Y., Tsai R. Y. (2013). Proc. Natl. Acad. Sci. U. S. A..

[cit46] Tsai R. Y., Kim S. (2005). J. Neurosci. Res..

[cit47] Tsai R. Y., McKay R. D. (2002). Genes Dev..

[cit48] Xiao D., Yan H., Wang Q., Lv X., Zhang M., Zhao Y., Zhou Z., Xu J., Sun Q., Sun K., Li W., Lu M. (2017). ACS Appl. Mater. Interfaces.

